# Perioperative prevention of euglycaemic diabetic ketoacidosis in people living with type 2 diabetes established on sodium-glucose transport-2 inhibitors – a cross-site multi-cycle audit

**DOI:** 10.1016/j.clinme.2025.100502

**Published:** 2025-08-12

**Authors:** Karl A. Romain, Jody Cheng, Seung Ho Luka Kim, Kenneth Watters, Aikaterini Theodoraki

**Affiliations:** a Department of Diabetes & Endocrinology, Chelsea & Westminster Hospital NHS Foundation Trust, 369 Fulham Road, London SW10 9NH, UK; b Imperial College London Faculty of Medicine, Exhibition Road, London SW7 2AZ, UK

**Keywords:** SGLT2 inhibitors, Perioperative prescribing, Preventing perioperative euglycaemic diabetic ketoacidosis

## Abstract

Sodium-glucose transport-2 (SGLT2) inhibitors are commonly prescribed for the management of type 2 diabetes mellitus, chronic kidney disease and heart failure. However, their continuation in the perioperative setting in people with diabetes can precipitate euglycaemic diabetic ketoacidosis (EDKA), a potentially life-threatening complication. This multi-cycle audit evaluated adherence to perioperative guidelines regarding preoperative cessation and postoperative re-initiation of SGLT2 inhibitors. Electronic health records of consecutive surgical patients with type 2 diabetes were reviewed over a 6-month period. Two cases of EDKA were identified. Targeted interventions – including staff education and dissemination of guidance – were implemented, followed by re-audit over a 4-month period. Documentation and perioperative SGLT2 inhibitor cessation improved significantly post-intervention; however, postoperative ketone monitoring remained suboptimal, and one further case of EDKA was identified. Since then, capillary blood ketone monitoring has been introduced in clinical areas. These findings add to the body of evidence on the perioperative use of SGLT2 inhibitors.

## Highlights

• Postoperative ketone monitoring is important to prevent euglycaemic diabetic ketoacidosis in patients living with diabetes established on SGLT2 inhibitors.
• Euglycaemic diabetic ketoacidosis events observed despite apparent normoglycaemia and protocol use.
• Education improved preoperative cessation documentation rates.

## Introduction

Sodium-glucose co-transporter-2 inhibitors (SGLT2i) are approved for the management of type 2 diabetes mellitus (T2DM), heart failure and chronic kidney disease.^1^^,^^2^ Their therapeutic benefit derives from inhibition of SGLT2-mediated glucose reabsorption in the proximal renal tubule, resulting in glucosuria and an osmotic diuresis. This mechanism contributes to reductions in blood pressure and intravascular volume.^3^

Perioperative continuation of SGLT2i is associated with an increased risk of euglycaemic diabetic ketoacidosis (EDKA), a rare but potentially fatal complication.^4^ Due to ongoing glucosuria, patients may develop ketoacidosis despite near-normal blood glucose levels, particularly in states of insulin deficiency or reduced oral intake.^5^ To mitigate against these risks, national guidelines recommend withholding SGLT2i preoperatively and reintroducing them cautiously once patients resume oral intake and ketone levels have normalised.^6^ In line with these, at Chelsea and Westminster Hospital NHS Foundation Trust, the local guideline advises withholding SGLT2i on the day before and the day of surgery, with re-initiation postoperatively once oral intake is re-established and ketone levels (either point-of-care or urinary) are within acceptable limits. Regular ketone monitoring is also advised following surgery. This retrospective audit was conducted to evaluate real-world adherence to these guidelines, identify gaps in practice and incidences of EDKA.

## Materials and methods

This was a clinical retrospective audit across the two trust hospital sites. We conducted a chart review of all surgical procedures (specialties included plastic surgery, general surgery, orthopaedic surgery, gynaecological surgery, urology) between 1 January and 30 June 2023, identifying patients with type two diabetes mellitus (T2DM) who were prescribed an SGLT2i at the time of surgery. A second cycle was conducted post-intervention between 15 November 2023 and 28 February 2024.

Electronic healthcare records (EHR) were searched. We filtered for patients who had a diagnostic code of type two diabetes mellitus (T2DM) and were prescribed a regular SGLT2i (canagliflozin, dapagliflozin, empagliflozin, ertugliflozin) at the time of surgery.

Baseline data included surgery type (elective or emergency), age, sex, hospital site, HbA1c, insulin prescription, postoperative day of SGLT2i recommencement, and type of SGLT2i. Outcomes were benchmarked against our trust guideline: appropriate preoperative cessation, ketone measurement before re-initiation, confirmation of oral intake, and EDKA incidence. For re-commencement, urinary or point-of-care ketones should be ≤ ++ or ≤ 0.6 mmol/L, respectively. EDKA was defined by pH < 7.30, bicarbonate < 18 mmol/L, ketosis (typically > 3 mmol/L) and glucose < 13.9 mmol/L.^7^

To assess cessation of SGLT2i preoperatively in elective cases, we looked at preoperative assessment documentation. For emergency cases, SGLT2i cessation was evaluated if the time interval from presentation to the procedure was permissive for the clinical team to withhold the medication. EHR were reviewed to ascertain whether ketones were recorded prior to SGLT2i recommencement and to note any instances of EDKA. To determine whether usual oral intake was established prior to recommencement, nursing and medical notes were reviewed.

Interventions included guideline promotion via email to the perioperative outpatient clinic and ward staff, dissemination of the audit findings via an internal audit presentation to our surgical pharmacists and preoperative teams, and posters with perioperative advice in clinical areas. For our statistical analysis we used Fisher’s exact test to compare categorical data. This included the binary answers to whether the medications were withheld and restarted correctly. An unpaired t-test was employed for normally distributed, continuous variables. This applied to patient characteristic data, such as age and HbA1c. For the event rate (events per surgical encounter) of EDKA, we calculated a binomial proportion 95% confidence intervals using the Clopper–Pearson exact test. All statistical tests were computed using the ‘DescTools’ package on ‘R’ and the significance for a type I error was set at p < 0.05.^8^

## Results

In the pre-intervention data set, 98 surgical procedures were identified. Post-intervention, 64 procedures were identified (Table 1).

**Table 1:** Baseline characteristics of the data sets (percentage in brackets unless specified).

	Elective pre-intervention (n = 44)	Elective post-intervention (n = 33)	Emergency pre-intervention (n = 46)	Emergency post- intervention (n = 29)
Age (years)	mean (SD) 65 (12)	mean (SD) 68 (11)	mean (SD) 65 (14)	mean (SD) 67 (12)
		p = 0.26		p = 0.53
Sex	n	n	n	n
Male	24 (55)	17 (52)	27 (59)	18 (62)
Female	20 (45)	16 (48) p = 0.82	19 (41)	11 (38) p = 0.81
Preoperative HbA1c (mmol/mol)	mean (SD)	mean (SD)	mean (SD)	mean (SD)
	61 (10)	55 (10) p = 0.01	68 (17)	61 (21) p = 0.12
Insulin dependent	n	n	n	n
Yes	11 (25)	11 (33)	11 (24)	11 (38)
No	33 (74)	22 (67) p = 0.45	35 (76)	18 (62) p = 0.21
POD SGLT2i recommencement	Mean (SD)	Mean (SD)	Mean (SD)	Mean (SD)
	2 (2)	2 (3) p = 1.00	5 (7)	3.2 (4) p = 0.21
SGLT2i Canagliflozin	n	n	n	n
	3 (7)	3 (9)	5 (11)	3 (10)
Dapagliflozin	24 (55)	18 (55)	15 (33)	19 (66)
Empagliflozin	17 (39)	12 (36)	26 (57)	7 (24)

SD, standard deviation; POD, postoperative day; SGLT2i, sodium-glucose transport-2 inhibitor; MEAN, arithmetic mean.

### Preoperative cessation

**Fig. 1:**
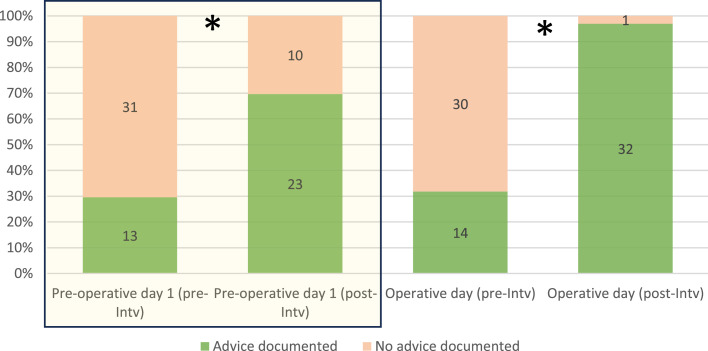
Percentage of pre-assessments in which SGLT2i cessation advice was documented. Absolute count in columns.

In the baseline audit, 30% of pre-assessments documented the appropriate withholding advice. Following the intervention, we noted a statistically significant improvement in the percentage of pre-assessments documenting cessation advice. A 40% improvement was recorded for advice concerning withholding the day prior to surgery (p < 0.001) and an over 65% improvement with regards to advice regarding withholding on the day of surgery (p < 0.001) (Fig. 1).

INTV., intervention; *, statistically significant difference after intervention.

**Fig. 2:**
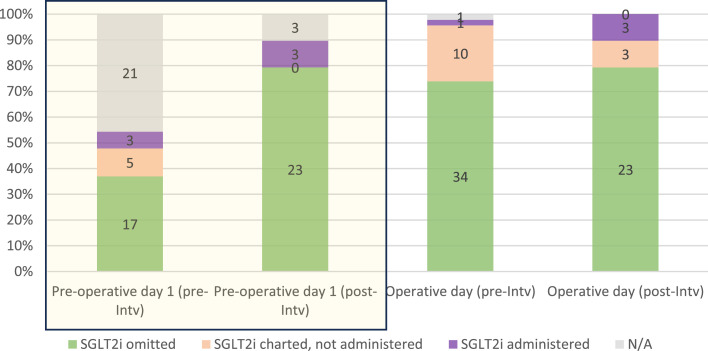
Percentage of emergency cases in which SGLT2i ommitted preoperatively. Absolute count in columns.

SGLT2i, sodium-glucose transport-2 inhibitor; N/A, not applicable; INTV., intervention; *, statistically significant difference after intervention.

In emergency procedures, 46% of cases could not be assessed for preoperative day 1 cessation due to the short time interval to surgery. In 32%, an SGLT2i on the day prior to surgery was prescribed, and in 12% of cases the SGLT2i was administered. Prescription and administration discrepancies were due to lack of medication availability on the wards or the patient having been made nil by mouth prior to their procedure. On the day of surgery, SGLT2i were withheld in 98% of cases. SGLT2i were charted in 24% of cases, but only administered in 2% (Fig. 2). Following our intervention, we identified an increase in the percentage withholding SGLT2i on the day prior to surgery, increasing from 68% to 88% (p = 0.076), and on the operative day, increasing from 76% to 79% (p = 0.245).

### Postoperative ketone measurement

**Fig. 3:**
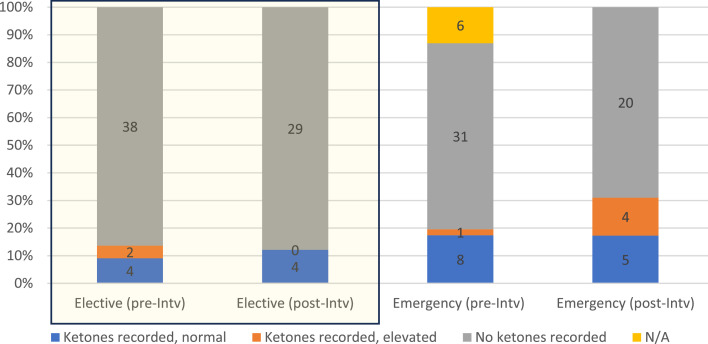
Percentage of cases in which postoperative ketone measurements performed. Absolute count in columns.

In six cases the SGLT2i was discontinued permanently, and the audit standard with regards to SGLT2i recommencement was not applicable. In 82% of cases, no postoperative ketone measurements were performed. When ketones were measured, three of 15 patients had ketones above the guidelines’ designated threshold at the time of recommencement (Fig. 3). Our results following intervention did not demonstrate an improvement.

N/A, not applicable; normal, with reference to the threshold for recommencement given in the local guidelines; INTV., intervention.

### Postoperative oral intake

In 90% of pre-intervention cases, patients established oral intake prior to SGLT2i recommencement. Following intervention, 89.5% of patients had documented established oral intake. Ketone monitoring, however, remained low, with only nine of 84 cases (11%) having documented both established normal oral intake and normal ketones pre-intervention. After intervention, seven of 57 cases (12%) fulfilled both criteria.

### Euglycaemic diabetic ketoacidosis

Three EDKA incidences were identified; two prior to the intervention and one following (Table 2). This corresponds to an event rate of 2.2% (95% CI: 0.27%, 7.80%) pre-intervention and 1.61% (95% CI: 0.04%, 8.66%) post-intervention (p = 1.00).

**Table 2:** Summary of EDKA cases.

Patient characteristics	76 female Emergency	71 male Elective	68 female Elective
Procedure	Dynamic right hip screw under spinal anaesthetic	Laparoscopic anterior resection for rectal carcinoma under GA	Laparoscopic extended right hemicolectomy
HbA1c, mmol/mol	80	49	67
Years since diabetes diagnosis	11	6	28
Regular diabetic medications	Canagliflozin 300 mg OD Metformin 1 g BD Gliclazide 160 mg BD	Dapagliflozin 10 mg OD Metformin 1 g BD Saxagliptin 5 mg OD Insulin isophane biphasic 22 u BD	Metformin 1 g BD Dapagliflozin 10 mg OD Insulin Lispro 10 u OD Insulin Glargine 30 u ON
Preoperative SGLT2i cessation	SGLT2i likely taken on day prior to op, as not admitted Withheld on day of procedure	No preoperative assessment advice documentation	Advised withholding on day
Diabetic medications on admission	No insulin intra-op, all diabetic medication discontinued	All diabetic medication discontinued. No VRII on admission	No insulin intra-op
Clinical course	NBM from admission. Blood glucose well controlled throughout admission (range: 5.4–12.4 mmol) On POD2 experienced persistent tachycardia VBG showed metabolic acidosis pH 7.28 Bicarbonate 13.1 mEq/L Glucose 11.2 mmol/L POC ketones 2.4 mmol/L Resolved with insulin infusion. Canagliflozin permanently discontinued.	NBM from admission. Uncomplicated 9 h long operation. Serial intra-op ABGs show worsening metabolic acidosis. pH 7.33 dropped to nadir of 7.17 post-operatively. Lactate always < 1.6mEq/L. Maximum glucose recording 10.7 mmol/L Lowest bicarbonate level 17.0 mEq/L. Transferred to intensive care. POC ketones 3.4 mmol/L Resolved within 24 h with insulin infusion. Patient re-commenced on dapagliflozin 10 days later, on discharge.	Postop reduced oral intake. No ketones measured. Restarted on dapagliflozin on POD3. Peri-arrest on POD5; patient found in metabolic acidosis pH 7.07, glucose 12.4 mmol/L, lactate 3.2 mEq/L, ketones 4.2 mmol/L. Resolved with insulin infusion.
Postoperative ketone measurements	Yes, after patient found to be acidotic		

POD, postoperative day; NBM, nil by mouth; ABG, arterial blood gas; VBG, venous blood gas; OD, once daily; BD, twice daily.

## Discussion

This retrospective two-cycle audit is a pragmatic evaluation of adherence to the perioperative use of SGLT2i recommendations, before and after a targeted intervention. Its strengths are the systematic review of consecutive series of patients with type 2 diabetes undergoing surgical procedures, including detailed review of electronic patient records, therefore reducing the risk of data loss and of misclassification of events. Our intervention significantly improved adherence to preoperative cessation advice in the elective setting but failed to improve postoperative ketone monitoring, which remained low ∼23%. One limitation of reviewing preoperative assessment documentation is that it was not possible to capture any written or verbal communication provided outside the medical records, therefore the adherence to SGLT2i cessation advice preoperatively may be underestimated.

We recognise that ketone testing was largely driven by symptomatic patients, and given the low rate of postoperative ketone testing, we risk having missed additional incidences of milder ketoacidosis. We did not audit ketosis in fasting patients who were not established on an SGLT2i, and a comparison group is thus lacking. We speculate that the uptake of perioperative ketone monitoring for patients on SGLT2i has increased with the introduction of point-of-care capillary ketone testing that has since become available locally in all clinical areas as of August 2024, and the associated blood ketone monitoring policy introduced. An electronic alert on prescribing SGLT2i, advising to ‘withhold in acute illness/starvation/perioperatively – can cause euglycaemic ketoacidosis’, has been in place since 2022.

In our series, EDKA occurred on postoperative days 1, 2 and 5; in the latter case, after restarting the SGLT2i. Increases in anion gap are acknowledged to occur in patients on SGLT2i when exposed to the physiological stress of surgery, and a strong association between decreased hold time and postoperative anion gap has been noted.^9^ Notably, EDKA occurred even with seemingly adequate glucose control and, in one case, appropriate preoperative SGLT2i cessation. All cases involved fasting, reduced insulin and prolonged perioperative drug exposure – known risk factors.^10^^,^^11^ While the perioperative cessation of SGLT2i for people with diabetes and ketone monitoring is expected to reduce the risk of EDKA, additional mitigation strategies may be required. In one multicentre prospective series of 759 patients with diabetes on SGLT2i, no cases of EDKA were observed perioperatively, which may be partly explained by the high rate of intraoperative glucose administration in 92.5%, and postoperative insulin at least once by postoperative day 3 in 58.5% of the patients.^12^ In contrast to UK guidelines, which recommend withholding the SGLT2i the day before and the day of surgical intervention preoperatively, the FDA recommends longer discontinuations times (3 days for dapagliflozin, empagliflozin and canagliflozin and 4 days for ertugliflozin).^13^^,^^14^

## Conclusion

This audit highlights ongoing risks associated with perioperative SGLT2i prescribing. Despite improvements in documentation, concordance with postoperative ketone monitoring remained low, reflecting systemic barriers such as unclear workflows. The lack of real-time ketone monitoring may have contributed to delayed diagnoses. The introduction of dual ketone–glucose monitors and implementation of a ketone monitoring protocol for high-risk patients aims to address this.

While improved documentation suggests increasing clinician awareness, emergency settings require streamlined prompts and integration into surgical workflows. Future efforts should focus on embedding decision aids into electronic records and reinforcing guideline adherence through automated alerts.

## CRediT authorship contribution statement

**Karl A. Romain:** Writing – review & editing, Writing – original draft, Visualization, Validation, Supervision, Software, Resources, Project administration, Methodology, Investigation, Formal analysis, Data curation, Conceptualization. **Jody Cheng:** Project administration, Methodology, Formal analysis, Data curation. **Seung Ho Luka Kim:** Validation, Project administration, Methodology, Investigation, Formal analysis, Data curation. **Kenneth Watters:** Validation, Supervision. **Aikaterini Theodoraki:** Writing – review & editing, Writing – original draft, Validation, Supervision, Resources, Project administration, Conceptualization.

## Declaration of competing interest

The authors declare that they have no known competing financial interests or personal relationships that could have appeared to influence the work reported in this paper.

## References

[1] Heerspink H.J.L., Stefánsson B V, Correa-Rotter R. (2020). Dapagliflozin in patients with chronic kidney disease. N Engl J Me.

[2] Dennis JM Henley WE McGovern AP et al. Time trends in prescribing of type 2 diabetes drugs, glycaemic response and risk factors: a retrospective analysis of primary care data, 2010–2017. *Diabetes, Obes Metab.* 2019 21 1576–1584 doi:10.1111/dom.13687. 10.1111/dom.13687 PMC6618851 30828962

[3] Fonseca-Correa J.I, Correa-Rotter R. (2021). Sodium-glucose cotransporter 2 inhibitors mechanisms of action: a review. Front Med.

[4] Somagutta M.R., Agadi K., Hange N. (2021). Euglycemic diabetic ketoacidosis and sodium-glucose cotransporter-2 inhibitors: a focused review of pathophysiology, risk factors, and triggers. Cureus.

[5] Mehta P.B., Robinson A., Burkhardt D., Rushakoff R.J. (2022). Inpatient perioperative euglycemic diabetic ketoacidosis due to sodium-glucose cotransporter-2 inhibitors – lessons from a case series and strategies to decrease incidence. Endocr Pract.

[6] Ayman G., Dhatariya K., Dhesi J. (2021).

[7] Long B., Lentz S., Koyfman A., Gottlieb M. (2021). Euglycemic diabetic ketoacidosis: etiologies, evaluation, and management. Am J Emerg Med.

[8] Signorell A DescTools: tools for descriptive statistics. 2025. Available from: https://cran.r-project.org/package=DescTools (Accessed August 19, 2025).

[9] Heijne A., Bronkhorst E.M. (2024). Dose-dependent relationship between SGLT2 inhibitor hold time and risk for postoperative anion gap acidosis. Comment on <em>Br J Anaesth</em>2023; 131: 682–686. Br J Anaesth.

[10] Steinhorn B., Wiener-Kronish J. (2023). Dose-dependent relationship between SGLT2 inhibitor hold time and risk for postoperative anion gap acidosis: a single-centre retrospective analysis. Br J Anaesth.

[11] Perry R.J., Rabin-Court A., Song J.D. (2019). Dehydration and insulinopenia are necessary and sufficient for euglycemic ketoacidosis in SGLT2 inhibitor-treated rats. Nat Commun.

[12] Seki H., Kuratani N., Shiga T. (2024). Incidence of sodium-glucose cotransporter-2 inhibitor-associated perioperative ketoacidosis in surgical patients: a prospective cohort study. J Anesth..

[13] Dhatariya K., Levy N., Russon K. (2024). Perioperative use of glucagon-like peptide-1 receptor agonists and sodium-glucose cotransporter 2 inhibitors for diabetes mellitus. Br J Anaesth..

[14] FDA Drug Safety FDA revises labels of SGLT2 inhibitors for diabetes to include warnings about too much acid in the blood and serious urinary tract infections available online: https://www.fda.gov/drugs/drug-safety-and-availability/fda-revises-labels-sglt2-inhibitors-diabetes-include-warnings-about-too-much-acid-blood-and-serious (Accessed 25 May 2015).

